# Touch Helps Hearing: Evidence From Continuous Audio-Tactile Stimulation

**DOI:** 10.1097/AUD.0000000000001566

**Published:** 2024-07-24

**Authors:** Xueying Fu, Fren T. Y. Smulders, Lars Riecke

**Affiliations:** 1 Faculty of Psychology and Neuroscience, Department of Cognitive Neuroscience, Maastricht University, Maastricht, the Netherlands.

**Keywords:** Auditory perception, Auditory processing, Electroencephalography, Steady-state response, Tactile

## Abstract

**Objectives:**

Identifying target sounds in challenging environments is crucial for daily experiences. It is important to note that it can be enhanced by nonauditory stimuli, for example, through lip-reading in an ongoing conversation. However, how tactile stimuli affect auditory processing is still relatively unclear. Recent studies have shown that brief tactile stimuli can reliably facilitate auditory perception, while studies using longer-lasting audio-tactile stimulation yielded conflicting results. This study aimed to investigate the impact of ongoing pulsating tactile stimulation on basic auditory processing.

**Design:**

In experiment 1, the electroencephalogram (EEG) was recorded while 24 participants performed a loudness-discrimination task on a 4-Hz modulated tone-in-noise and received either in-phase, anti-phase, or no 4-Hz electrotactile stimulation above the median nerve. In experiment 2, another 24 participants were presented with the same tactile stimulation as before, but performed a tone-in-noise detection task while their selective auditory attention was manipulated.

**Results:**

We found that in-phase tactile stimulation enhanced EEG responses to the tone, whereas anti-phase tactile stimulation suppressed these responses. No corresponding tactile effects on loudness-discrimination performance were observed in experiment 1. Using a yes/no paradigm in experiment 2, we found that in-phase tactile stimulation, but not anti-phase tactile stimulation, improved detection thresholds. Selective attention also improved thresholds but did not modulate the observed benefit from in-phase tactile stimulation.

**Conclusions:**

Our study highlights that ongoing in-phase tactile input can enhance basic auditory processing as reflected in scalp EEG and detection thresholds. This might have implications for the development of hearing enhancement technologies and interventions.

## INTRODUCTION

Detecting and selecting target sounds, particularly within challenging noisy surroundings, constitutes a fundamental aspect of our everyday experiences. Performance in this task can be improved by nonauditory stimuli. A common example is the practice of lip-reading, which can enhance both the cortical representation and the intelligibility of speech in noise (Sumby & Pollack 1954; Driver 1996; Calvert et al. 1997; Park et al. 2016). While the influence of visual input on auditory processing has been demonstrated in many studies, the widespread use of face masks during the coronavirus disease 2019 pandemic prevented lip-reading and underscored the need to explore also potential auditory benefits from other, nonvisual information (Ramdani et al. 2022). The processing of somatosensory (tactile) stimulation exhibits notable similarities with the processing of auditory stimulation in terms of both physiological mechanisms and developmental trajectories (see Occelli et al. 2011 for a comprehensive review). In our daily life, we can catch an incoming ringtone easily when the vibration function on the phone is enabled; nevertheless, our knowledge of how tactile stimuli affect auditory processing remains relatively limited.

Audio-tactile research has often used brief tactile stimuli (shorter than 500 msec) and consistently found a facilitating effect on auditory perception (Schürmann et al. 2004; Murray et al. 2005; Gillmeister & Eimer 2007; Gick et al. 2008; Gick & Derrick 2009; Wilson et al. 2009; Reed et al. 2021). These studies have emphasized the crucial role of the simultaneity of the onsets of auditory and tactile stimuli for auditory perception. A possible interpretation is that salient onsets/offsets of the brief tactile stimuli temporarily elevated listeners’ vigilance and thereby improved their auditory performance (Arrabito et al. 2015; Al-Shargie et al. 2019). More direct auditory benefits from tactile stimulation, beyond those mediated by onset/offset-related vigilance, can be studied using longer-lasting, ongoing tactile stimulation that lacks salient onsets/offsets. However, audio-tactile studies using longer-lasting audio-tactile stimulation so far have obtained conflicting results in both linguistic/music and basic auditory research, with some studies confirming tactile effects on auditory perception (Timora & Budd 2013; Huang et al. 2017; Fletcher et al. 2018, 2019; Cieśla et al. 2019, 2022; Aker et al. 2022; Guilleminot & Reichenbach 2022; Schulte et al. 2023; Liapikou & Marozeau et al. 2024) and others finding such effects only at the neural level (Timora & Budd 2018; Riecke et al. 2019; Fu & Riecke 2023). The inconsistencies observed in linguistic studies might be attributed to various potential factors such as training, hearing impairment, language familiarity, and semantic predictability, which are beyond the scope of the present study. While benefits for auditory perception and speech intelligibility have been observed with short tactile pulse sequences, to the best of our knowledge, no evidence thus far has demonstrated the facilitation of basic auditory perception by continuous, ongoing tactile stimulation that lacks salient, distinct onsets/offsets. The latter was the focus of the present study. If ongoing tactile stimulation lacking salient onsets/offsets can produce auditory benefits, it might allow supporting normal or hearing-impaired listeners over long periods in an ongoing manner and possibly without distracting them from the auditory input. In real-life listening tasks, this would require instantaneous extraction of onsets/offsets within the auditory stream of interest to allow defining the ongoing tactile stimulation protocol.

Regarding the neural basis of auditory benefits from tactile stimulation, Timora and Budd conducted a series of electroencephalography (EEG) studies (Timora & Budd 2013, 2018) to explore the influence of 1.5-sec long tactile stimulation on auditory sensitivity. Specifically, they presented amplitude-modulated auditory stimuli with or without simultaneous amplitude-modulated tactile stimulation and measured amplitude-modulation (AM) detection thresholds. Contrary to their initial expectations, the results revealed a decrease in auditory sensitivity when tactile stimulation was introduced, despite an enhancement in the auditory cortical response (Budd & Timora 2013; Timora & Budd 2013, 2018). Similar neural outcomes were obtained in a study conducted within our laboratory (Fu & Riecke 2023). In that study, continuous auditory noise and tactile stimulation with the same 4-Hz AM rate were presented either in-phase or anti-phase. Participants were asked to ignore the tactile stimulation and discriminate a brief loudness decrease of a continuous auditory signal (37-Hz) embedded in the noise. Neuroelectric responses to the auditory noise were found to be enhanced by the in-phase tactile stimulation, and responses to the auditory signal were suppressed by the anti-phase tactile stimulation. However, no corresponding effect on perceptual performance could be observed. One possible explanation for this discrepancy is that participants did not pay attention to the modulation frequency of the tactile stimulation, thereby limiting its impact on auditory perception. This possibility is partly supported by previous observations of a facilitating effect of attention on auditory perception and multisensory processing (Fritz et al. 2007; Talsma et al. 2010; Macaluso et al. 2016; Lage-Castellanos et al. 2023). Another potential explanation could be the use of an inadequate task paradigm. The tactile stimulation was designed to affect the masking potential of the auditory noise, and this was measured with a loudness-discrimination task on a tone in the noise. Thus, this task provided only an indirect measure of the hypothesized auditory effect, possibly rendering this measure too insensitive.

In the present study, we aimed to investigate these possibilities and characterize the effect of ongoing periodic tactile input on auditory processing, as well as the putative role of attention in this. We presented a 4-Hz AM tone-in-noise and ongoing 4-Hz AM tactile stimulation that was either in-phase or anti-phase with the AM tone. In experiment 1, we recorded EEG while participants performed a loudness-discrimination task on the tone. However, unlike in the previous study (Fu & Riecke 2023), the sound that co-fluctuated with the 4-Hz tactile stimulation was critical for participants’ task and therefore it required the participants’ focus of attention. In experiment 2, participants underwent the same tactile stimulation as participants in experiment 1, but performed a tone-in-noise detection task. This task required them to pay selective attention to their left or right ear and detect brief occurrences of the tone-in-noise, which was presented to either the attended or the unattended ear. In line with previous results (Budd & Timora 2013; Timora & Budd 2013, 2018; Fu & Riecke 2023), we expected that the in-phase tactile input would enhance the neuroelectric response to the tone whereas the anti-phase tactile input would suppress it. More importantly, in experiment 1, we hypothesized that these neural effects would be paralleled by corresponding effects on the audibility of the tone-in-noise and therewith on the detectability of the tone-loudness changes. That is, we expected that in-phase tactile input would facilitate auditory perception while anti-phase tactile input would hamper it, because the tactile input fluctuated at the same rate as the tone. In experiment 2, we expected to find similar tactile-induced effects on the detectability of the tone in the noise and an additional facilitating effect of selective auditory attention.

## EXPERIMENT 1

### Method

#### Stimuli

The stimuli were similar to those in Fu and Riecke (2023). They were generated in MATLAB (The MathWorks, Natick, MA) with a sampling rate of 16 kHz and transformed to analog signals using a multichannel D/A converter (National Instruments).

**Fig. 1.**
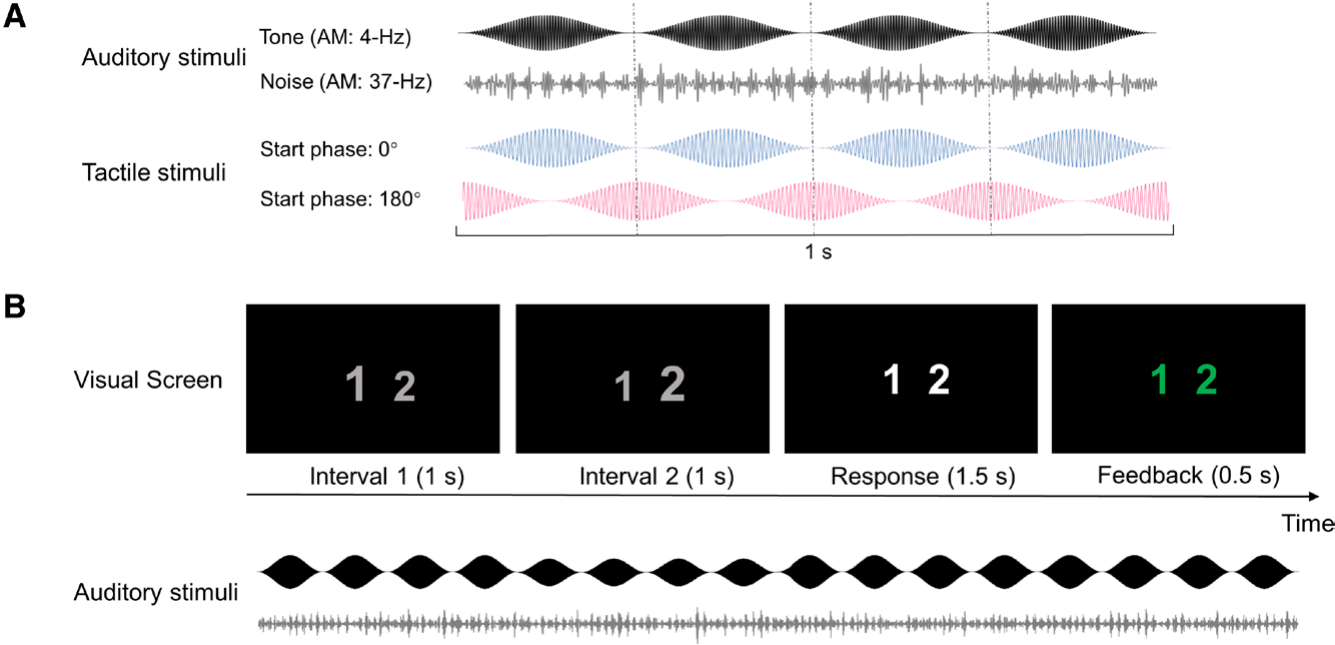
Stimuli and experimental design of experiment 1. A, Auditory and tactile stimuli. The black and gray waveforms show the auditory tone (amplitude modulation at 4 Hz) and noise (amplitude modulation at 37 Hz), respectively. The blue and magenta waveforms show respectively the in-phase and anti-phase tactile stimulation, which had the same 4-Hz amplitude modulation as the tone. The audio-tactile phase shift was achieved by adjusting the start phase of the tactile stimulation to be either 0° or 180° relative to the initial phase of the auditory tone. B, Example of a trial when the target occurred during the 2nd interval. During each trial, two consecutive observation intervals were visually indicated on a screen by the continuously displayed digits “1” and “2,” which temporarily increased in size for the duration of the observation interval. The target, a 1-sec decrease in the loudness of the continuous tone, occurred during either the 1st or 2nd interval. The participants’ task was to detect this decrease and indicate the interval during which the decrease occurred by pressing the corresponding button “1” or “2” during the response interval. Following each trial, visual feedback regarding response correctness was presented. The tone and noise were played continuously during the entire block, the SNR and the type of stimulation (audio-tactile in-phase, audio-tactile anti-phase, or auditory only) were varied across blocks. SNR indicates signal to noise ratio.

The auditory stimuli consisted of a fluctuating tone and a fluctuating noise that were presented simultaneously and continuously during stimulation blocks of 4 min (as shown in Fig. 1A). The tone was a 250-Hz sinusoid carrying an amplitude modulation of 4 Hz (sinusoidal modulation, depth: 100%, start phase: 0°). It was presented at a fixed sound-pressure level of 44 dB SPL, which we deemed sufficiently high for evoking measurable auditory scalp potentials and sufficiently low for preventing participant discomfort from auditory stimulation. The intensity of the tone was reduced by 3 dB during brief intervals of 1 sec to define an auditory target. The onsets and offsets of the tone and the target were ramped within 10 msec. The noise was generated from a Gaussian distribution and band-pass filtered to a 1-octave range centered on 250 Hz (4th order Butterworth filter with zero phase shift). The noise was amplitude-modulated at 37 Hz (sinusoidal modulation, depth: 80%, start phase: 0°) and presented at various intensities resulting in four signal to noise ratios (SNRs): 9.3, 4.9, 1.4, and −1.5 dB. This SNR manipulation was included to allow exploring the hypothesized tactile effect at various performance levels and support participants’ motivation (by inducing perceptual variability and avoiding prolonged exposure to overly easy or challenging listening conditions). Auditory stimuli were delivered diotically using insert earphones (EARTone 3A) in a sound-attenuating chamber.

The tactile stimuli consisted of an alternating electric current resembling a 175-Hz sinusoid that carried an amplitude modulation of the same frequency as the amplitude modulation of tone (4 Hz, sinusoidal modulation, depth: 100%, start phase: 0° or 180°, which is in-phase or anti-phase with the tone modulation). Tactile stimuli were delivered transcutaneously and inaudibly to the participant via an isolated bipolar constant-current stimulator (Digitimer DS5, Digitimer, UK) controlled by the D/A converter and two rubber electrodes (3 × 3 cm^2^), which were placed over the peripheral median nerve on the right forearm. The current amplitude was set individually using an adjustment procedure, ensuring that the tactile stimulation was perceivable, tolerable, and non-painful for each participant (range across participants: 0.8 to 3.9 mA, mean [*M*] ± SD: 1.6 ± 0.7 mA; peak-to-zero amplitude).

#### Experimental Design and Data Recordings

The design was defined by fully crossing the two factors stimulation and SNR. The stimulation included three different types of stimuli: auditory-alone stimulation (condition A), in-phase audio-tactile stimulation (AT_0_), and anti-phase audio-tactile stimulation (AT_180_). The SNR factor comprised the four levels of the auditory noise. Stimulation and SNR were fixed during each block and each Stimulation × SNR combination (i.e., condition) was presented once, resulting in twelve unique blocks that were presented in individually randomized order.

Auditory perception was evaluated using a two-interval two-alternative forced-choice (2I2AFC) tone-loudness-discrimination task. Each trial commenced with the display of two consecutive 1-sec observation intervals, visually indicated by numbers (Fig. 1B). The target was randomly presented in either the first or the second interval with equal probability across trials. Participants were instructed to direct their attention toward the tone, ignore both auditory noise and tactile input (if present), and identify the specific interval containing the target by pressing the corresponding button during a 1.5-sec response interval after the second observation interval. Visual feedback on response correctness was given after each response. In total, 720 trials were presented (60 per condition). Visual stimuli were presented and behavioral responses were recorded using Presentation software (version 20.0, Neurobehavioral Systems, Inc., Albany CA), which was controlled by the D/A converter and MATLAB. Before the main measurement, participants were familiarized with the stimuli and practiced the task in two mini-blocks (20 trials per mini-block). They were not provided with any explicit instruction or training pertaining to the integration of auditory and tactile stimuli, nor were they given guidance on distinguishing the relative phase of audio-tactile stimulation.

During the task, EEG was recorded at a sampling rate of 1000 Hz via a BrainAmp amplifier (Brain Products, Munich, Germany) and 30 scalp Ag/AgCl electrodes: Fz, FCz, Cz, CPz, Pz, POz, FP1/FP2, F3/F4, F7/F8, FC1/FC2, FC5/FC6, C3/C4, T7/T8, CP1/CP2, CP5/CP6, P3/P4, P7/P8, O1/O2. One additional electrode was placed on the right mastoid (M1), and another electrode was placed below the left eye to record the vertical EOG. Electrode impedance was kept under 5 kΩ. At the beginning and end of the EEG recording, an additional 4-min block of tactile-alone stimulation was presented to allow exploring potential changes in neural responsiveness to the tactile stimulation over the duration of the experiment. At the end of the session, participants’ ability to identify the in-phase versus anti-phase audio-tactile stimulation (AT_0_ versus AT_180_) was assessed using an audio-tactile synchrony-judgment task (similar to Fu & Riecke 2023). This one-interval two-alternative forced-choice task involved 15 AT_0_ trials and 15 AT_180_ trials in random order. On each trial, a single AT stimulus (duration: 5.5 sec including 1-sec on/off ramps) was presented. Participants were required to focus simultaneously on both sensory input modalities and judge whether they pulsated in-phase or in an alternating fashion. Visual feedback on response correctness was presented after each response.

#### Participants

Twenty-eight healthy adults (11 males and 17 females), with ages ranging from 19 to 34 years were recruited. All participants had normal hearing (pure-tone audiometric thresholds <25 dB HL for 250 to 8000 Hz in both ears), reported no relevant medical conditions (i.e., no skin condition, epilepsy, neurological, or psychiatric disorder), and had not taken part in our previous audio-tactile study (Fu & Riecke 2023). Written informed consent was obtained, and the experimental procedure was approved by the local ethics committee. Four participants were excluded based on predefined criteria (see Data analysis), consequently, 9 males and 15 females (*M* ± SD: 21.9 ± 3.5 years) were included in the final analysis.

### Data Analysis

#### Behavioral Data

Behavioral data were analyzed using MATLAB (2020a; The MathWorks, Inc.) and SPSS (SPSS Statistics Version 28). To assess individual discrimination performance, accuracy was computed by dividing the number of correct responses by the number of trials, for each of the twelve conditions. A ceiling effect was operationally defined as an accuracy above 90% in condition A at the lowest SNR, and a floor effect as an accuracy below the chance level (50%) in condition A at the highest SNR. One participant was excluded because of a floor effect.

#### EEG Preprocessing

EEG data were analyzed with the EEGLAB 14.1.2 toolbox (Delorme & Makeig 2004) and custom scripts in MATLAB. The EEG channel data were re-referenced to the average of all scalp electrodes. Independent component analysis was used to reduce artifacts, with a band-pass filter (cutoff frequencies: 1 and 40 Hz) applied to the continuous data beforehand. Artifactual components were identified by visual inspection and removed. The remaining nonartifactual components were applied to the original unfiltered data, which were then band-pass filtered using wider cutoff frequencies (0.5 and 45 Hz) (Jaeger et al. 2018). Figure 1 in Supplemental Digital Content, http://links.lww.com/EANDH/B469, shows that the band-pass filtering effectively removed electric artifacts caused by the tactile stimulation. Vertical EOG and M1 channels were not included in subsequent EEG data analyses. The artifact-reduced data were segmented into 1-sec epochs and baseline-corrected, more details are described in Fu and Riecke (2023). Artifact reduction based on independent component analysis and subsequent application of a 75-µV amplitude criterion resulted in the rejection of on average 6.1% ± 3.3% artifactual components and 2.1% ± 2.5% artifactual epochs (*M* ± SD across participants). Two participants were excluded due to excessive EEG artifacts (more than 15% of EEG epochs in any condition). On average, 1645 ± 42 epochs per participant were retained for further analysis.

#### SSR Magnitude

Steady-state responses (SSRs) were assessed to evaluate the magnitude of phase-locked cortical responses to the fluctuating sensory inputs. This involved averaging EEG epochs across all observation intervals in the time domain per condition, followed by a discrete Fourier transform with 1000 data points, resulting in a spectral resolution of 1 Hz. The magnitude of the SSR was determined by dividing power at the stimulation frequency (“tagging frequency,” 4 or 37-Hz) by the noise floor, which was computed as the average power of the two frequency bins neighboring the tagging frequency. SSR values were expressed in decibels (dB). These analytical procedures were conducted independently for each EEG channel, experimental condition, and participant.

To explore the impact of the tactile stimulation on neuroelectric responses to the tones, we first selected those EEG channels that reliably showed a strong 4-Hz response in the auditory-alone, highest-SNR condition and then evaluated if the average response of these channels was modulated by the tactile stimulation. We chose the aforementioned condition for the channel selection because we expected it to elicit the clearest and strongest auditory tone-evoked responses, which we deemed to reflect auditory processing. The channels were selected at the group level by statistically comparing the SSR versus zero with a one-tailed paired *t* test and correcting the resulting channel-wise *p*-values using the false discovery rate. Only channels with a corrected *p*-value <0.05 were selected. The selected channels were Fz, FCz, Cz, POz, F3/F4, FC1/FC2, FC5/FC6, C3/C4, T7/78, CP6, P3/P4, P7/P8, and O1/O2. The same approach was used to extract neuroelectric responses to the auditory noise, with the only difference that the 37-Hz SSR was extracted from the auditory-alone, lowest SNR condition; this analysis resulted in the selection of all channels.

#### Statistical Testing

Observations that deviated from the distribution mean by more than three SDs were identified as outliers and excluded from the analysis, which concerned one participant. To assess the assumption of normality, Kolmogorov–Smirnov tests were conducted. No significant deviations from normality were detected. Mauchly tests were conducted to assess the assumption of sphericity, and when the assumption was violated, Greenhouse–Geisser correction was applied to adjust the degrees of freedom.

Two-way repeated-measures analysis of variances (ANOVAs), including the three stimulation conditions (condition A, AT_0_, and AT_180_) and four SNRs as factors, were applied to test the effects of stimulation and SNR on behavioral and EEG data. In addition, one-tailed simple contrasts were used to investigate the enhancement induced by AT_0_ and the suppression caused by AT_180_ in relation to the auditory-alone condition (A). Multiple linear regression was performed to assess the relationship between neuroelectric responses and behavioral performances. A significance level of *p* < 0.05 was applied to all tests. Partial eta-squared (*η*^2^_*p*_) was used to quantify effect sizes.

### Results

#### Behavioral Results

**Fig. 2.**
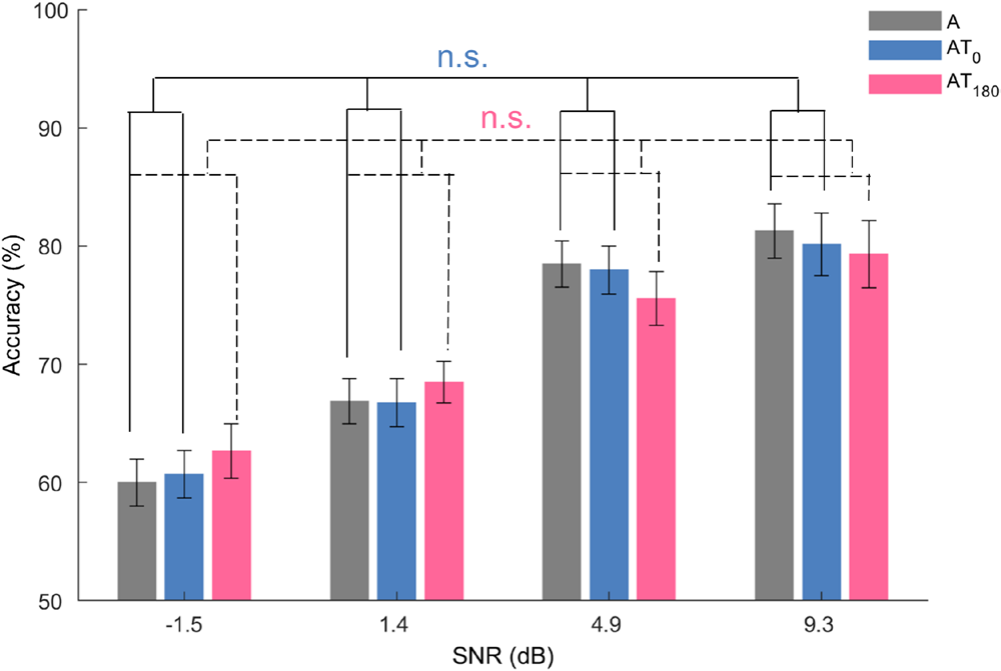
Behavioral results of experiment 1. The bar plot illustrates the average accuracy of listeners across the different stimulation conditions as a function of SNR. The baseline performance, represented by the gray bars, pertains to the auditory-alone condition (A), while the blue and magenta bars correspond to the in-phase audio-tactile condition (AT_0_) and anti-phase audio-tactile condition (AT_180_), respectively. Data show the M ± SE across participants. Above the lines, n.s. represents no significant difference between two stimulation conditions. SNR indicates signal to noise ratio.

A three (stimulation: condition A, AT_0,_ and AT_180_) by four (SNR: −1.5, 1.4, 4.9, and 9.3 dB) ANOVA on accuracy was conducted to investigate the impact of tactile stimulation on auditory perception. The results (as shown in Fig. 2) confirmed a significant main effect of SNR [*F*(3,69) = 92.60, *p* < 0.001, *η*_*p*_^2^ = 0.80], showing better discriminability when the SNR was higher. In contrast with our hypothesis, we found no main effect of stimulation [*F*(2,46) = 0.02, *p* = 0.979, *η*_*p*_^2^ = 0.001] nor an interaction [Stimulation × SNR: *F*(6,138) = 0.91, *p* = 0.491, *η*_*p*_^2^ = 0.04], indicating that the presence of tactile input did not significantly affect auditory perception. Further examination of the differences between the auditory-alone condition and audio-tactile conditions using simple contrasts revealed no significant difference [AT_0_ versus A: *F*(1,23) = 0.06, *p* = 0.402, *η*_*p*_^2^ = 0.003; AT_180_ versus A: *F*(1,23) = 0.01, *p* = 0.460, *η*_*p*_^2^ < 0.001]. These simple contrasts also showed no significant interaction Stimulation × SNR (*P*s > 0.089). These results provided no evidence for an influence of the ongoing tactile input on auditory performance in the discrimination task.

Participants’ audio-tactile synchrony-judgments showed an accuracy of 48.8% ± 10.4% (mean ± SD; range: 30 to 73.3%), which did not differ significantly from the chance level of 50% (one-tailed paired *t* test: *t* [23] = 0.59, *p* = 0.718). This indicates that participants could not reliably identify the relative phase of the audio-tactile stimuli.

#### 4-Hz Steady-State Response

**Fig. 3.**
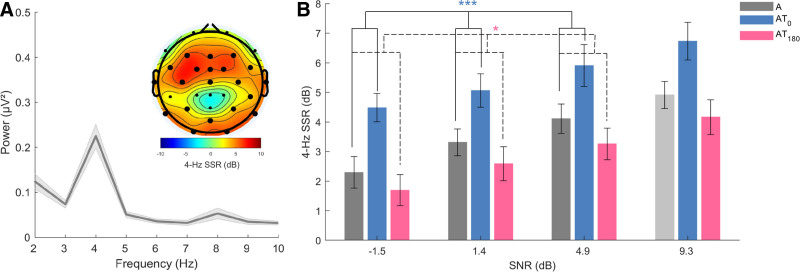
EEG power spectrum and topographical distribution of the 4-Hz SSR response to the tone (left) and the channel-averaged 4-Hz SSR (right). A, The topographic map shows the average 4-Hz auditory SSR in the auditory-alone condition at the highest SNR. This map was deemed to reflect the cortical representation of the 4-Hz amplitude modulation of the tone and was used to select channels at which the 4-Hz auditory SSR was significantly above zero (*p* < 0.05, FDR-corrected, enlarged dots). Consequently, the EEG power spectrum averaged across the selected channels shows a stronger response at 4 Hz than at neighboring frequencies. B, The channel-averaged 4-Hz SSR for each stimulation condition as a function of SNR. Solid lines and dashed lines refer respectively to the main effect of stimulation for A vs. AT_0_ and A vs. AT_180_. Above the lines, *** and * represent *p* < 0.001 and *p* < 0.050, respectively. Condition A at the highest SNR is plotted in light gray because its value is biased by the prior channel selection, which was based on that condition. EEG indicates electroencephalogram; FDR, false discovery rate; SSR, steady-state response.

Initial inspection of neural responses to the tactile stimulation before and after the auditory experiment suggested that tactile-evoked responses were relatively stable and did not change significantly over the duration of the experiment (Figure 2 in Supplemental Digital Content, http://links.lww.com/EANDH/B469). To investigate the neural processing of the auditory stimulation, we first extracted phase-locked neuroelectric responses to the tone. We selected EEG channels with a significant 4-Hz SSR in the auditory-alone condition at the highest SNR in the tone. The scalp topography of the 4-Hz auditory SSR and the selected channels are shown in Figure 3A, showing that the tone-evoked response was most prominent over fronto-central regions.

To investigate the impact of tactile inputs on the tone-evoked response, a two-way repeated-measures ANOVA with factors of stimulation (condition A, AT_0_, and AT_180_) and SNR (−1.5, 1.4, and 4.9 dB) was applied to the channel-averaged 4-Hz SSR. Main effects of SNR [*F*(2,46) = 9.52, *p* < 0.001, *η*_*p*_^2^ = 0.29] and stimulation [*F*(2,46) = 26.62, *p* < 0.001, *η*_*p*_^2^ = 0.54] were observed, but no interaction [*F*(2.75,63.14) = 0.16, *p* = 0.910, *η*_*p*_^2^ = 0.01]. Further examination of the differences between auditory-alone condition and audio-tactile conditions using simple contrasts revealed a significant difference between condition AT_0_ and condition A [*F*(1,23) = 24.32, *p* < 0.001, *η*_*p*_^2^ = 0.51], showing that the in-phase tactile input significantly enhanced the response to the tone, compared with the auditory-alone input. Conversely, the comparison between condition AT_180_ and condition A revealed a significant difference in the opposite direction [*F* (1,23) = 4.95, *p* = 0.018, *η*_*p*_^2^ = 0.18], indicating that the anti-phase tactile input reduced the tone-evoked response, compared with the auditory-alone input. Also with these simple contrasts, no significant interaction Stimulation × SNR could be observed (*P*s > 0.448). These results suggest that the neuroelectric response to the auditory tone was modulated by the tactile stimulation, and the direction of this effect depended on the audio-tactile phase.

#### 37-Hz Steady-State Response

**Fig. 4.**
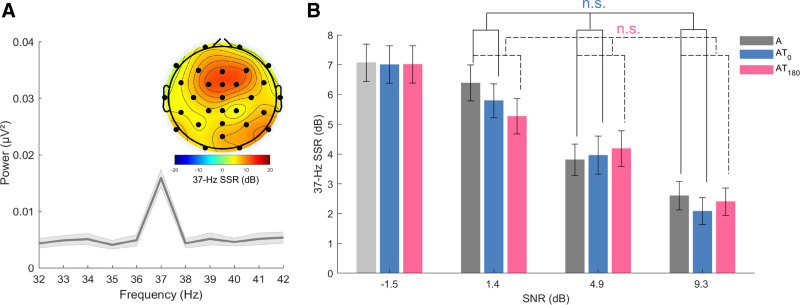
Neuroelectric representation of the amplitude modulation of the auditory noise (left) and the channel-averaged 37-Hz SSR (right). Same as Figure 3 but for 37-Hz SSR. SSR indicates steady-state response.

The analysis that was applied to the 4-Hz SSR was also applied to the 37-Hz SSR (as shown in Fig. 4) to investigate whether the tactile stimulation also modulated responses to the noise, which fluctuated at an unrelated rate.

A two-way ANOVA confirmed a significant main effect of SNR on the 37-Hz SSR [*F*(2,46) = 75.57, *p* < 0.001, *η*_*p*_^2^ = 0.77]. However, no significant main effect of stimulation or interaction was detected (*F*s < 0.88, *P*s >.357, *η*_*p*_^2^ < 0.04). These results suggest that the 4-Hz tactile input did not affect the processing of the 37-Hz AM noise.

### Interim Discussion

In sum, the findings from experiment 1 demonstrate that tactile input can affect EEG responses to auditory input fluctuating at the same rate, and the direction of this effect (enhancement versus suppression) depends on the relative phase of the audio-tactile input. However, despite the observation of a tactile influence on neuroelectric responses and the fact that participants paid attention to auditory input fluctuating at the same rate as the tactile input, no corresponding tactile effect on auditory perception could be observed. A possible explanation for this behavioral null result may be a lack of sensitivity of our measure of auditory perception, which was based on performance in a 2I2AFC loudness-discrimination task. More specifically, according to the inverse effective principle, an auditory enhancement from tactile stimulation is more pronounced at lower sound levels. Therefore, such enhancement would be expected to affect the (softer) target interval more strongly than the other (louder) observation interval, thereby reducing their discriminability. To explore this idea, we further analyzed loudness discrimination at the neural level, that is, differences between neural responses to the two observation intervals. This yielded a similar pattern of null results as the behavioral analyses above (Figures 3 and 4 in Supplemental Digital Content, http://links.lww.com/EANDH/B469). These supplementary results suggest that any tactile effect on tone loudness in experiment 1 affected both observation intervals similarly, making it difficult to detect any tactile effect on the discrimination of these two intervals. On the basis of this, behavioral effects of tactile stimulation on auditory perception were further addressed in experiment 2 with a different, nondiscriminative behavioral paradigm.

## EXPERIMENT 2

### Method

#### Stimuli

A yes/no task requiring participants to detect a tone-in-noise was used to assess the effect of tactile stimulation on auditory perception. Auditory noise and tactile stimulation were the same as in experiment 1, with the only difference that the intensity of the noise was fixed at 65 dB SPL. The tone had a duration of 750 msec (corresponding to three 4-Hz cycles, including 10 msec cosine-squared on/off ramps) and was presented monaurally at either 0° (AT_0_) or 180° (AT_180_) of the 4-Hz tactile input at a sound-pressure level corresponding to one of the four following SNRs: −20, −16, −12, and −8 dB. Auditory stimuli were presented via a pair of headphones (Sennheiser HD650). The intensity of the tactile stimulation was on average 1.4 ± 1.0 mA (*M* ± SD across participants, range: 0.6 to 5.2 mA, peak-to-zero amplitude).

Auditory spatial attention was directed to either the left or the right ear in different blocks of trials. This was done by displaying three visual arrows throughout each block centrally on a black background on an LCD screen via Presentation software (Neurobehavioral Systems, Inc.) controlled by MATLAB. The tone was presented to the requested ear with a probability of 75% and to the nonrequested ear with a 25% probability. At the beginning of each trial, the arrows were shown in dark gray to indicate a wait interval. During a subsequent pretone interval, the arrows turned light gray to indicate the upcoming target interval. To reduce the risk that the onsets of the visual cue could induce valid temporal predictions about the exact target onset or systematic oscillatory phase resets (Elliott et al. 2010; Fiebelkorn et al. 2011; Daume et al. 2021), the duration of the pretone interval was pseudorandomly varied across trials (uniform distribution from 0 to 490 msec). The duration of the wait interval was adjusted accordingly, so that the overall duration from trial onset to tone onset corresponded to either 500, 625, 750, 875, or 1000 msec (mean: 750 msec). This latter variation across trials served to implement the audio-tactile phase manipulation and further reduce the temporal predictability of the tone.

#### Experimental Design and Procedure

**Fig. 5.**
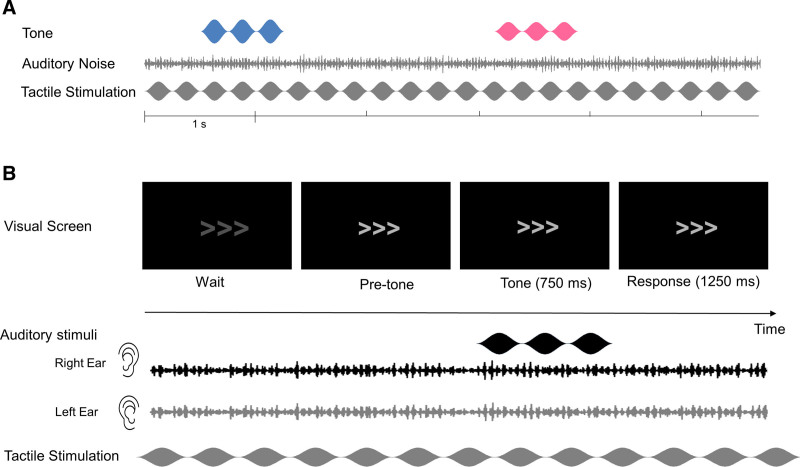
Stimuli and task design of experiment 2. A, Example of two consecutive trials including in-phase and anti-phase audio-tactile stimulation. The top waveform illustrates the target, which was a tone with sinusoidal amplitude modulation at 4 Hz. The blue and magenta waveforms show a target at a high SNR that is in-phase with the tactile stimulation (relative phase is 0°) and a target at a lower SNR that is in anti-phase with the tactile stimulation (relative phase is 180°), respectively. The middle waveform shows the continuous auditory noise with a sinusoidal amplitude modulation at 37 Hz and fixed overall intensity. The bottom waveform shows the continuous tactile stimulation with sinusoidal amplitude modulation at 4 Hz. B, Example of a trial when participants were asked to focus on the right ear. The arrows were shown on a screen continuously during the whole block to indicate to the participant the to-be-attended ear. Dark gray arrows illustrated the beginning of each trial, and the tone (duration: 750 msec) was presented with a variable delay after the arrows turned light gray. Continuous auditory noise was presented diotically, while the target was presented monaurally at either the attended ear (75%) or unattended ear (25%). Participants were required to press a button corresponding to “Tone” or “No Tone” as accurately as possible. SNR indicates signal to noise ratio.

The experiment used a balanced 3 (stimulation: A, AT_0_, and AT_180_; see Fig. 5A) × 4 (SNR: −20, −16, −12, and −8 dB) × 2 (attention: attended, unattended) factorial within-subject design. While the auditory-alone condition and audio-tactile condition were presented in separate blocks, the in-phase audio-tactile and anti-phase audio-tactile conditions were presented randomly within audio-tactile blocks. Each block consisted of 120 trials, of which 96 contained a tone presented to a given ear (target-present trials) and 24 contained no tone presented to any ear (target-absent trials) in random order. Within the 96 target-present trials, each of the four SNRs was randomly presented 24 times. Moreover, of these 96 target-present trials, 72 trials (75%) involved presentation of the tone to the to-be-attended ear, and 24 trials (25%) involved presentation of the tone to the to-be-unattended ear. The latter 3:1 ratio served to encourage participants to focus their attention on the requested, to-be-attended ear. Overall, the experiment consisted of twelve blocks that included four blocks of auditory-alone stimulation (two blocks with attention to each ear) and eight blocks of audio-tactile stimuli (four blocks with attention to each ear), lasting in total approximately 66 min. The order of blocks was individually randomized.

Participants were instructed to press one of two buttons (Fig. 5B) corresponding to “Tone” or “No Tone” as accurately as possible within 2 sec after the onset of the tone (if present), after which the arrows on the screen immediately turned dark gray. Before the main measurement, participants practiced the task and received trial-based visual feedback on response correctness in two mini-blocks (40 trials per mini-block). One of these practice blocks involved the auditory-alone condition and attention to the left or right ear, whereas the other block involved an audio-tactile condition and attention to the other ear.

#### Participants

Data were collected from 28 university students. Four participants were excluded from further analysis because of a technical issue (defect headphone cable), a poor psychometric fit (goodness-of-fit [GOF] <0.70, see Psychometric function), or an outlier threshold (>3 SD away from the mean). Ultimately, data from 24 participants (5 males and 19 females; mean age: 20.3 ± 1.9 years) were used for statistical analyses. None of the participants had a history of hearing or ear-related problems, skin diseases, or neurological or psychiatric disorders based on self-reports. None of them had participated in the previous audio-tactile study (Fu & Riecke 2023) or experiment 1. All participants had normal and symmetric hearings, defined as pure-tone audiometric thresholds of less than 25 dB HL at 250, 500, 750, 1000, 2000, and 4000 Hz in both ears, and an interaural threshold difference of less than 10 dB at each frequency.

### Data Analysis

In each stimulation condition, the proportion of affirmative (“Tone”) responses was computed separately for target-present trials and target-absent trials to obtain hit rates and false-alarm rates, respectively.

#### Psychometric Function

Hit rates per SNR were modeled individually for each stimulation condition using a logistic psychometric function in MATLAB, using the formula $y=1/\left(1+e^{-\sigma \left(x-\mu \right)}\right)$, where x represents the SNR, μ indicates the SNR yielding 50% accuracy on the psychometric function (i.e., the threshold), and σ determines the slope of the psychometric function. Individual psychometric curves are displayed in Figures 5–7 in Supplemental Digital Content, http://links.lww.com/EANDH/B469. The GOF of the psychometric function was measured by $R^{2}$. Only individual psychometric functions showing a satisfactory fit ($R^{2}$ > 0.70) were analyzed; the GOF of these functions was observed to range from 0.82 to 1.00 (*M* ± SD: 0.97 ± 0.05). Threshold was estimated from the fitted function and subsequently statistically compared across conditions.

#### Statistical Testing

The frequency distributions of thresholds were normally distributed in all conditions. Two-way repeated-measures ANOVA, with stimulation (A, AT_0_, and AT_180_) and attention (attended, unattended) as factors, was applied to test the potential impact of phasic tactile input on the detection threshold, as well as the potential influence of attention on this putative effect. Paired *t* tests were conducted to assess the difference in false alarms between auditory-alone and audio-tactile conditions. Cohen *d* was used as an effect size estimator for *t* tests.

### Results

**Fig. 6.**
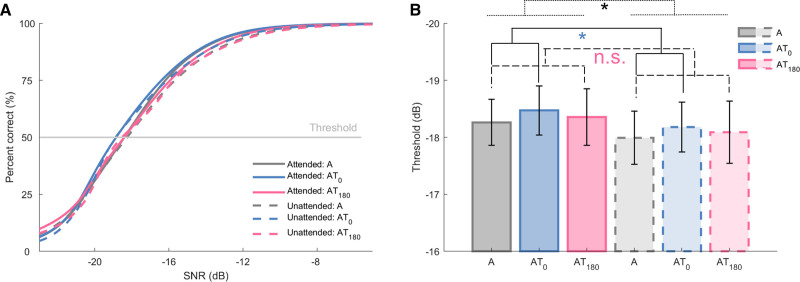
Behavioral results of experiment 2. A, Psychometric curves showing the percentage of correct responses as a function of SNR in the attended condition (soiled lines), unattended condition (dashed lines) for the auditory-alone condition (A, gray), in-phase audio-tactile condition (AT_0_, blue) and anti-phase audio-tactile condition (AT_180_, magenta). The curves were obtained by averaging the individual curves shown in Figures 5–7 in Supplemental Digital Content, http://links.lww.com/EANDH/B469. B, The average threshold across participants. Bars (error bar: SE) with solid lines and dashed lines show threshold in the attended and unattended condition, respectively. Below the lines, * and n.s. represent *p* < 0.05 and no significant main effect, respectively. SNR indicates signal to noise ratio.

Figure 6 depicts the fitted curves representing behavioral accuracy as a function of SNR. A three (stimulation: condition A, AT_0_, and AT_180_) by two (attention: attended, unattended) ANOVA was conducted to explore the effects of tactile stimulation and attention on the tone-detection threshold estimated from the psychometric function. No interaction of Stimulation × Attention [*F*(1.4,31.4] = 0.004, *p* = 0.982, *η*_*p*_^2^ < 0.001] or significant main effect of stimulation [*F*(1.4,32.2) = 0.58, *p* = 0.506, *η*_*p*_^2^ = 0.03] was observed, but a significant main effect of attention [*F*(1,23) = 6.46, *p* = 0.018, *η*_*p*_^2^ = 0.22], indicating that the auditory threshold was lower in the attended condition compared with the unattended condition. We then further investigated simple effects of tactile stimulation in simple contrast analyses. Comparison of in-phase audio-tactile versus auditory-alone conditions revealed a significant effect of in-phase stimulation on threshold [main effect of stimulation, AT_0_ versus *A*: *F*(1,23) = 3.20, *p* = 0.043, *η*_*p*_^2^ = 0.12], in line with our hypothesis. That is, in-phase tactile stimulation reduced the auditory threshold, reflecting an improvement in auditory perception. No significant effect of anti-phase tactile stimulation on threshold was observed [no main effect of stimulation, AT_180_ versus A: *F*(1,23) = 0.22, *p* = 0.320, *η*_*p*_^2^ = 0.01] and no interaction Stimulation × Attention was found in either analysis (*P*s > 0.923). These results suggest that both in-phase tactile input and selective auditory attention improved auditory perception.

To rule out the possibility that the observed effect of tactile input reflected a mere response bias (i.e., an increased willingness of participants to report the target, regardless of their auditory perception), false alarms were compared between the auditory-alone and audio-tactile conditions. In target-absent trials, participants’ responses were pooled across attention conditions and phase conditions as both variables were defined relative to the target (i.e., these variables were only meaningful when the target was actually present). Paired *t* tests revealed no statistically significant difference in false alarms between the two conditions (*M* ± SD of condition A: 6.7% ± 6.0%; *M* ± SD of condition AT: 7.2% ± 7.3%; *t* [23] = 0.40, *p* = 0.695, *d* = 0.08), suggesting that the tactile stimulation did not merely increase the number of target reports. This further suggests that the observed effect on threshold reflects an improvement in auditory perception rather than a response bias.

In sum, the findings from experiment 2 demonstrate that both ongoing in-phase tactile input and selective auditory attention can improve auditory performance in a tone-in-noise detection task.

## DISCUSSION

In this study, we investigated the impact of continuous tactile input on the cortical processing and perception of an AM tone-in-noise, with a focus on the effects of the audio-tactile phase and the listener’s selective attention. In experiment 1, scalp-recorded SSRs confirmed that in-phase audio-tactile input, compared with auditory-alone input, can enhance the response to the fluctuations of the tone, while anti-phase audio-tactile input can suppress it. However, no corresponding effect on behavioral loudness discrimination could be found. In experiment 2, a yes/no paradigm revealed that in-phase audio-tactile input, but not anti-phase audio-tactile input, can enhance the perceptual detection of a tone-in-noise. Overall, these findings demonstrate that ongoing tactile input can influence neuroelectric and perceptual aspects of basic auditory processing, with a relevant role of the audio-tactile phase.

### Ongoing Tactile Input Can Affect Auditory Perception

Our primary finding is that continuously ongoing tactile stimulation can have a small, but significant benefit for basic auditory perception. In experiment 1, we initially assessed auditory perception through a loudness-discrimination task. In this task, participants were required to detect a brief intensity reduction of a continuous tone-in-noise. Unexpectedly, we found no tactile stimulation effect on behavioral performance in this task, despite the observation that in-phase tactile input significantly enhanced the neuroelectric response to the tone. While this pattern of results confirms our prior study (Fu & Riecke 2023), it suggests that paying attention to auditory fluctuations that are tightly coupled with tactile stimulation is insufficient to elicit a tactile effect on auditory perception (note that participants in the previous study performed the same discrimination task on a tone that fluctuated independently of the tactile stimulation). A more plausible explanation for the behavioral null result is the discriminative nature of the loudness-discrimination task. Our examination of the difference in 4-Hz SSR between no-target and target intervals (delta 4-Hz SSR) indicated that this difference indeed correlated significantly with listeners’ loudness-discrimination performance and remained essentially unaffected by tactile input (refer to Figures 3 and 4 in Supplemental Digital Content, http://links.lww.com/EANDH/B469). Put differently, the ongoing tactile input exhibited similar effects in the two consecutive intervals, implying that it could not directly enhance any processing difference between these two intervals, explaining the null-effect on performance.

On the basis of these initial results, and previous ideas about different psychophysical measures for audio-tactile perception (Timora & Budd 2013), we assessed tactile effects on auditory perception in experiment 2 with a tone-in-noise detection task that required no discrimination of observation intervals. Instead, listeners simply detected a tone in continuous noise, with or without continuous tactile input. In contrast with the behavioral null result from experiment 1, we found an auditory benefit: in-phase audio-tactile inputs led to lower thresholds compared with purely auditory input, and this effect was independent of auditory spatial attention. To our knowledge, these results show for the first time that ongoing in-phase tactile input can enhance basic aspects of auditory perception, in line with our hypothesis and previous studies using brief tactile pulses (Schürmann et al. 2004; Gillmeister & Eimer 2007; Wilson et al. 2009, 2010), as well as some linguistic studies using sequential tactile input (Cieśla et al. 2022; Guilleminot & Reichenbach 2022). An analysis of false-alarm rates indicated that the observed tactile effect on threshold did not reflect a response bias. This result provides support for the notion that the in-phase tactile stimulation primarily affected participants’ auditory perception rather than their response strategy. Overall, these results suggest that in-phase tactile input can facilitate auditory perception in an ongoing manner.

In contrast with the facilitating effect of in-phase tactile stimulation, the anti-phase tactile stimulation had no significant impact on tone-in-noise detection, suggesting that ongoing anti-phase tactile input does not influence auditory perception. This lack of a tactile effect is consistent with several reports in the literature (Fu & Riecke 2023; Gillmeister & Eimer 2007; Riecke et al. 2019; but see Guilleminot & Reichenbach 2022). It is worth noting that the response enhancement of in-phase tactile input in experiment 1 was consistent with the perceptual facilitation observed in experiment 2, while no corresponding perceptual suppression was observed with anti-phase audio-tactile inputs. This discrepancy may be attributed to differences in the strength of the neuroelectric effect of these two types of stimulation: the in-phase tactile stimulation enhanced the 4-Hz SSR by on average 2 dB, whereas the anti-phase tactile stimulation reduced it by only 0.7 dB. It is possible that behavioral changes require a sufficiently robust modulation of brain responses.

### Ongoing Anti-phase Tactile Input Can Suppress Neuroelectric Responses to Sound

In experiment 1, we investigated the impact of continuous tactile input on auditory responses using the frequency-tagging technique as in previous studies (Budd & Timora 2013; Timora & Budd 2013, 2018; Fu & Riecke 2023). We observed that in-phase 4-Hz tactile inputs enhanced 4-Hz SSR, but not 37-Hz SSR, compared with auditory-alone input, showing that ongoing fluctuating tactile input can boost the scalp response to in-phase auditory stimuli. This result aligns well with previous neural findings (Foxe et al. 2002; Murray et al. 2005; Hoefer et al. 2013; Riecke et al. 2019; Guilleminot & Reichenbach 2022; Fu & Riecke 2023) and parallels our behavioral results in experiment 2.

Conversely, we observed that anti-phase 4-Hz audio-tactile input reduced the 4-Hz SSR, but not 37-Hz SSR, indicating that ongoing fluctuating tactile input can also inhibit the scalp response to auditory stimuli when they are anti-phase. In our previous study (Fu & Riecke 2023), we also observed a suppressive effect of anti-phase 4-Hz tactile stimulation. However, unlike the present results, this affected only responses to a sound that fluctuated at a tactile-unrelated rate (37-Hz), not responses to a sound at the tactile-related 4-Hz rate. This might be related to the fact that only the 37-Hz sound was task-relevant. Together, the anti-phase effects observed in the two studies suggest that anti-phase tactile stimulation affects responses to sounds that are task-relevant, even when they fluctuate at a tactile-unrelated rate, whereas responses to task-irrelevant sounds remain relatively unaffected.

This possibility is partly supported by Misselhorn et al. (2016), in which participants were required to attend to two out of three sensory modalities and perform a congruence-matching task. Poorer matching performance was observed when incongruent stimulation occurred in the attended versus unattended modality, suggesting that attention amplified the influence of incongruent stimulation on perception. Thus, differences in the task relevance of the tactile stimulation rate or top-down attention toward versus away from the tactile modulation rate might explain the observed difference in neural results between our two studies. Alternatively, they might be explained by “bottom-up” acoustic factors, such as differences in carrier sound (tone versus noise) or modulation rate (4 versus 37 Hz). The SSR indeed has been shown to be sensitive to top-down factors (Müller et al. 2009; Bharadwaj et al. 2014; Manting et al. 2020) as well as acoustic factors (Picton et al. 1987; Lins et al. 1996).

Our observation that ongoing tactile input can exert facilitative as well as suppressive neuroelectric effects (depending on the audio-tactile phase) confirms the coexistence of facilitation and suppression effects that has been found by previous EEG studies on multisensory processing (Talsma & Woldorff 2005; Wang et al. 2012). Specifically, the facilitation effect has been shown to occur in an early time window (~130 msec after stimulus onset), which may reflect the rapid integration of multisensory information and the amplification of neural activity when congruent or complementary sensory signals are present. In contrast, the suppression effect tends to manifest in a later time window (~200 msec after stimulus onset), which may reflect the resolution of conflicting or incongruent sensory inputs, leading to a top-down regulation of neural activity to optimize perceptual processing (Wang et al. 2012). Our neural measure (the SSR) integrates neural responses over a time scale of multiple seconds, prohibiting analysis of the aforementioned specific time windows. However, it is conceivable that the in-phase stimulation activated predominantly the aforementioned early process, whereas anti-phase stimulation activated more the later process. Subsequent studies are needed to address the temporal dynamics underlying the different effects of ongoing in-phase and anti-phase audio-tactile stimulation.

### Limitations

While our study reveals auditory benefits from in-phase audio-tactile stimulation, it is essential to acknowledge that the observed reduction in the tone-in-noise detection threshold was relatively moderate, amounting to less than a 1 dB difference. This limitation could potentially be attributed to the absence of extensive participant training in our study, as audio-tactile training has been shown to yield significant improvements in for example, speech recognition (Cieśla et al. 2019, 2022).

In addition, although we focused the analysis on EEG channels that showed the strongest auditory responses, it remains possible that these channels reflected not only auditory processing, but also processing of the tactile input. This ambiguity arises from the fact that neuroelectric signals recorded from the scalp are inherently nonspecific due to the phenomenon of volume conduction, making it difficult to conclusively attribute them to for example, auditory or somatosensory cortical sources (Özcan et al. 2005; Rodgers et al. 2008). Animal studies using invasive neuroelectric methods with higher spatial resolution have shown that tactile input can modulate sound-evoked responses in the auditory cortex (Kayser et al. 2005; Lakatos et al. 2007), suggesting that our scalp-levels measures at least partially reflect such modulations.

Noteworthy, we observed the enhancing effects of in-phase tactile input on neuroelectric responses and perceptual performance in separate experiments with different participants. This difference precludes establishing a direct link between these observations and leaves unclear whether the enhancing effects on neuroelectric and perceptual measures reflect the same underlying process. Future studies could address this limitation by measuring EEG and tone-in-noise detection performance simultaneously in the same participants.

## CONCLUSION

This study provides evidence that ongoing periodic tactile input can exert influences on neuroelectric and perceptual aspects of basic auditory processing, with an important role of the relative phase of the audio-tactile inputs. The findings may inform the development of touch-based technologies for hearing enhancement and interventions in individuals with hearing impairments.

## ACKNOWLEDGMENTS

The authors thank Emily Bendlin for assistance with data collection and all the members of the Auditory Perception and Cognition section for valuable suggestions and discussions.

## Supplementary Material


