# Supplementary material for: Touch Helps Hearing: Evidence From Continuous Audio-Tactile Stimulation
Source: Ear Hear. 2024 Jul 24;46(1):184–95. doi: 10.1097/AUD.0000000000001566 (PMC11637573; doi:10.1097/AUD.0000000000001566)
Supplement: Supplementary file 1 [file aud-46-184-s001.pdf]

Supplementary Material

EEG power spectrum during tactile stimulation

Supplementary Figure 1 below shows the EEG power spectrum (averaged across electrodes and participants) during tactile stimulation before bandpass filtering of the EEG recordings (panel A) and after filtering (panel B). Data from auditory-alone stimulation and in-phase audio-tactile stimulation are shown for reference. In the raw data, the tactile stimulation induced an artifact at the carrier frequency of the tactile stimulation (175Hz) and its sidebands (175 ± 4Hz, induced by the 4-Hz amplitude modulation). The filtering (see section 2.2.2, *EEG data preprocessing*) effectively removed this artifact, resulting in flat spectra in the aforementioned frequency range in all conditions.

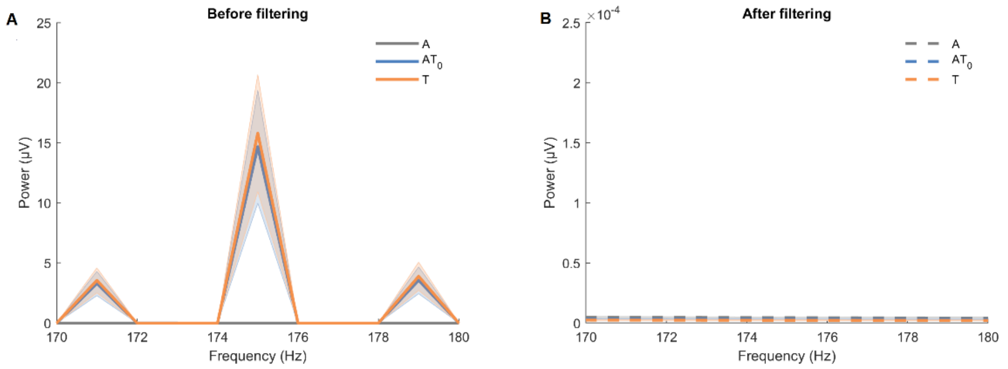

Supplementary Figure 1. *The average EEG power spectrum was recorded in the absence of tactile stimulation (A) and in the presence of tactile stimulation (AT_0 and T).*

A: *The spectrum from 170 Hz to 180 Hz before bandpass filtering of the raw EEG recordings. The gray waveform shows the spectrum in the absence of tactile stimulation (purely auditory condition, lowest SNR), which essentially overlaps with the x-axis at the shown scale. Blue and orange waveforms show spectra during audio-tactile stimulation (in-phase audio-tactile condition, AT_0, at the lowest SNR) and tactile-alone stimulation, revealing clear peaks at the carrier frequency of the tactile stimulation (175 Hz) and its sidebands (175 Hz ± 4 Hz, reflecting the 4-Hz modulation of the carrier).*

B: *Same as panel A, but after bandpass filtering of the raw EEG recordings. Contrary to the left panel, spectral peaks are no more observable (the three spectra essentially overlap with the x-axis), indicating that the bandpass filtering effectively reduced the tactile stimulation artifacts. Note the different power scales in the two panels.*

4-Hz SSR to tactile stimulation

To verify that effects of tactile stimulation on cortical activity were stable for the duration of the experiment, an additional 4-minute block of tactile-alone stimulation was presented at the beginning and end of the EEG experiment. A similar analysis as the main analysis was applied to extract the cortical response to tactile stimulation, that is, the 4-Hz tactile SSR was extracted and based on its significance, channels were selected (Fz, FCz, Pz, FP1/FP2, F3/F4, FC1/FC2, CP1, CP5, P3, P7, and O1). The average topographical distribution of the tactile SSR is illustrated below, showing that tactile responses were on average most prominent over the frontocentral and contralateral scalp regions, which may reflect respectively premotor / supplementary motor cortex (Scrivener & Reader, 2022) and parietal (supramarginal area/ second somatosensory area) cortex, in line with earlier tactile studies (Allison et al., 1992; Bottini et al., 1995). As can be seen in Figure S2, tactile responses near the end of the experiment were slightly weaker than those at the beginning. However, no significant difference was observed between the two measurements (t (1,23) = 1.70, p = .102) and the order of conditions in the EEG experiment was randomized across participants, suggesting that the cortical response to tactile stimulation was relatively stable across the experiment and could not systematically confound the main results reported in the manuscipt.

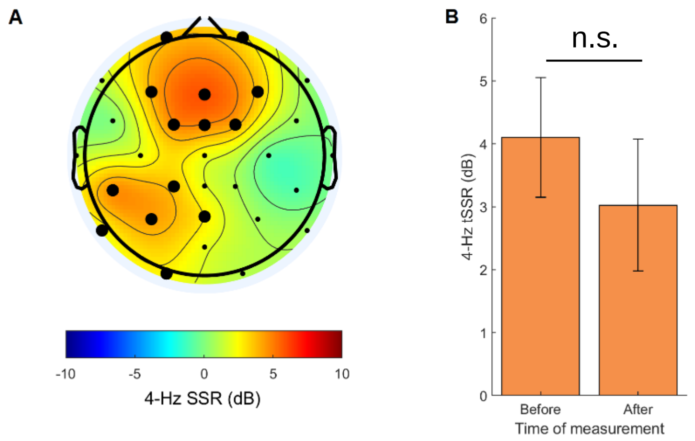

Supplementary Figure 2. *Average 4-Hz tactile SSR recorded before and after the main EEG experiment.*

A: *The topographic map shows the average 4-Hz SSR to purely tactile stimulation. Black dots represent scalp locations of the EEG electrodes, and enlarged dots represent locations at which the 4-Hz tactile SSR was significantly above zero (p < 0.05, FDR-corrected).*

B: *The bar plot shows the channel-averaged 4-Hz tactile SSR measured before and after the main EEG experiment (Error bar: SE). Above the line, n.s. represents no significant difference.*

Comparison of cortical responses to target vs. no-target

Considering the discriminative nature of the 2I2AFC task, we also analyzed the difference in the 4-Hz SSR during no-target intervals vs. target intervals (SSR during no-target interval minus SSR during target interval; referred to as “delta 4-Hz SSR”). We observed a significantly positive delta 4-Hz SSR in every condition, indicating that the target (a loudness decrease) reliably reduced the 4-Hz SSR as expected. However, we found no main effect of *Stimulation* on delta 4-Hz SSR, nor an interaction. Overall, the pattern was very similar to that of the behavioral (null) results (cf. main Figure 2).

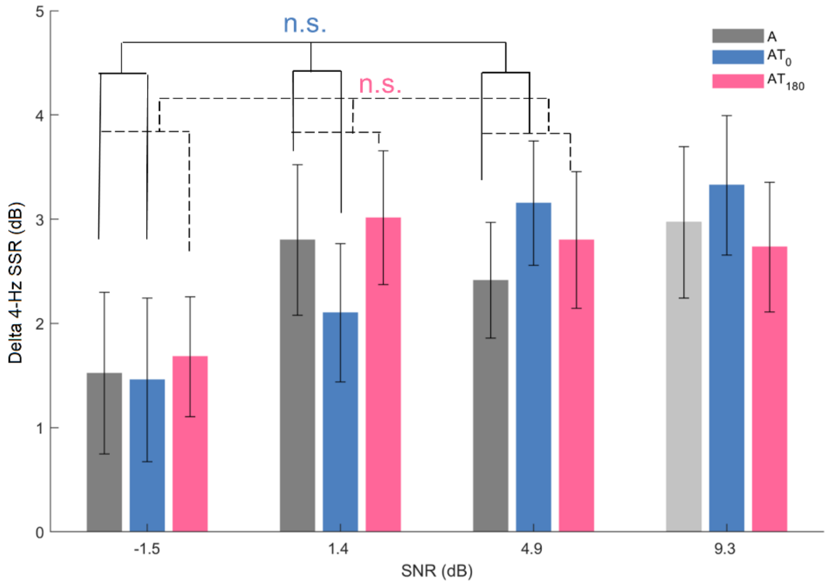

Supplementary Figure 3. *Cortical 4-Hz response magnitude difference (delta 4-Hz SSR) between no-target and target interval in each condition. The bar plot shows the difference in the average 4-Hz SSR between no-target intervals and target intervals for each stimulation condition as a function of SNR. Positive values indicate that the occurrence of the target reduced the 4-Hz SSR, reflecting the loudness decrease of the tone. The gray bars, blue bars, and magenta bars show data from the purely auditory condition (A), in-phase audio-tactile condition (AT_0) and anti-phase audio-tactile condition (AT_{180}), respectively. Condition A at the highest SNR is shown in light gray as this data point may be biased due to the fact that it rpartially defined the selected channels. Data show the mean ± SE across participants. Solid lines and dashed lines refer respectively to the main effect of Stimulation for A vs. AT_0 and A vs. AT_{180}. Below the lines, n.s. represents no significant main effect.*

Relation between behavioral performance and 4-Hz SSR

To explore a potential lack of sensitivity of our measure of auditory perception, we further investigated the relationship between neural loudness discrimination (delta 4-Hz SSR) and perceptual performance in the 2I2AFC tone-loudness discrimination task with a multiple linear regression analysis. Behavioral accuracy was treated as the response variable, whereas *Stimulation*, *SNR* and delta 4-Hz SSR were treated as predictor variables. Accuracy and SSR were pooled across *Stimulation* and *SNR* conditions, as there was no significant interaction observed between *Stimulation* and *SNR* for either measure.

The fit of the overall regression model was significant (F (3, 284) = 49.38, p < .001, R^2 = 0.343). Among the predictor variables, *SNR* and delta 4-Hz SSR exhibited significant positive effects on accuracy (*SNR*: β = .018, t (284) = 11.23, p < .001; delta 4-Hz SSR: β = .006, t (284) = 2.98, p = .003), whereas *Stimulation* had no effect (β = -.001, t (284) = -0.15, p = .880). Further analysis revealed that the inclusion of delta 4-Hz SSR as a predictor in the regression model improved its explanatory power by 2.1 percentage points. These findings suggest that beyond the influence of *SNR*, the individuals' neural representation of tone loudness (as measured with the delta 4-Hz SSR) contributed to their performance in the loudness discrimination task.

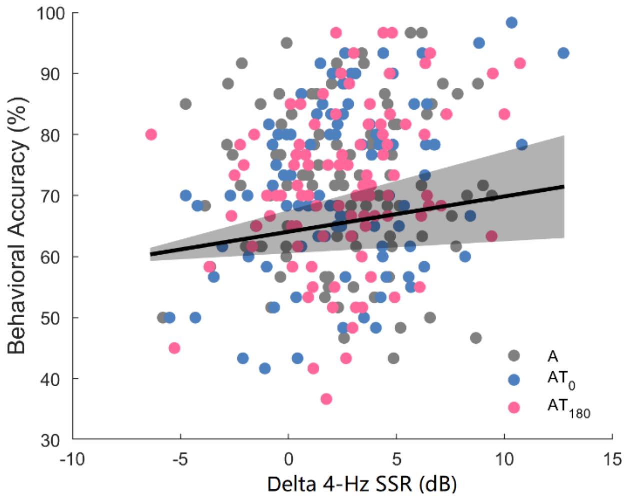

Supplementary Figure 4. *Scatterplot of accuracy and delta 4-Hz SSR (calculated by subtracting the 4-Hz SSR to target intervals from the 4-Hz SSR to no-target intervals). Gray dots represent individual participants in Condition A. Observations from the same participants in conditions AT_0 and AT_{180} are shown respectively in blue and magenta. The black line illustrates the positive linear relationship between participants' overall accuracy and delta 4-Hz SSR according to the multiple linear regression. The shaded area represents the 95% confidence interval of the regression model.*

Individual psychometric curves

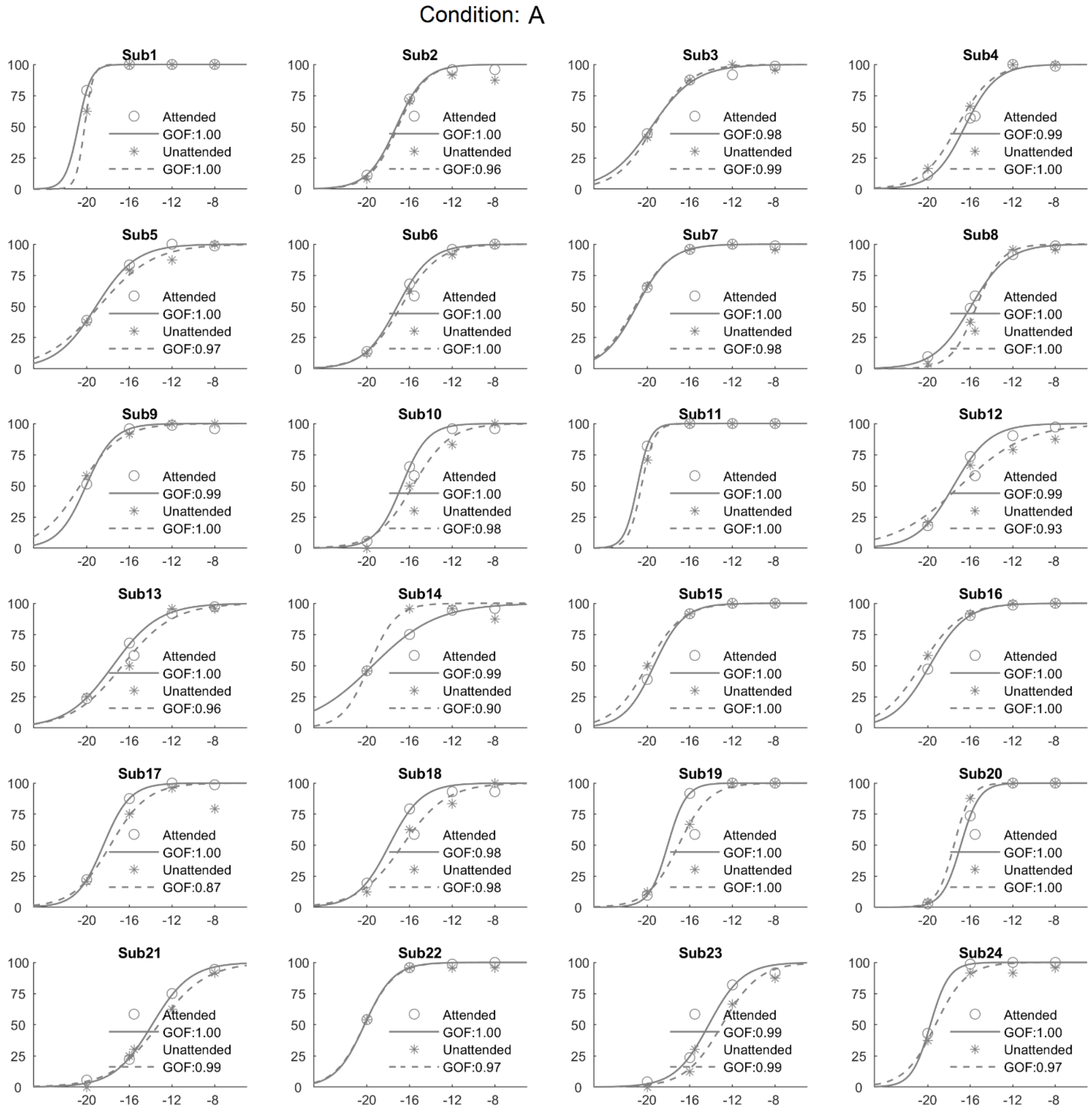

Supplementary Figure 5. *The percentage of correct responses as a function of SNR for each participant in the auditory-alone stimulation condition. The solid line and dashed line represent psychometric curves in the attended and unattended conditions, respectively.*

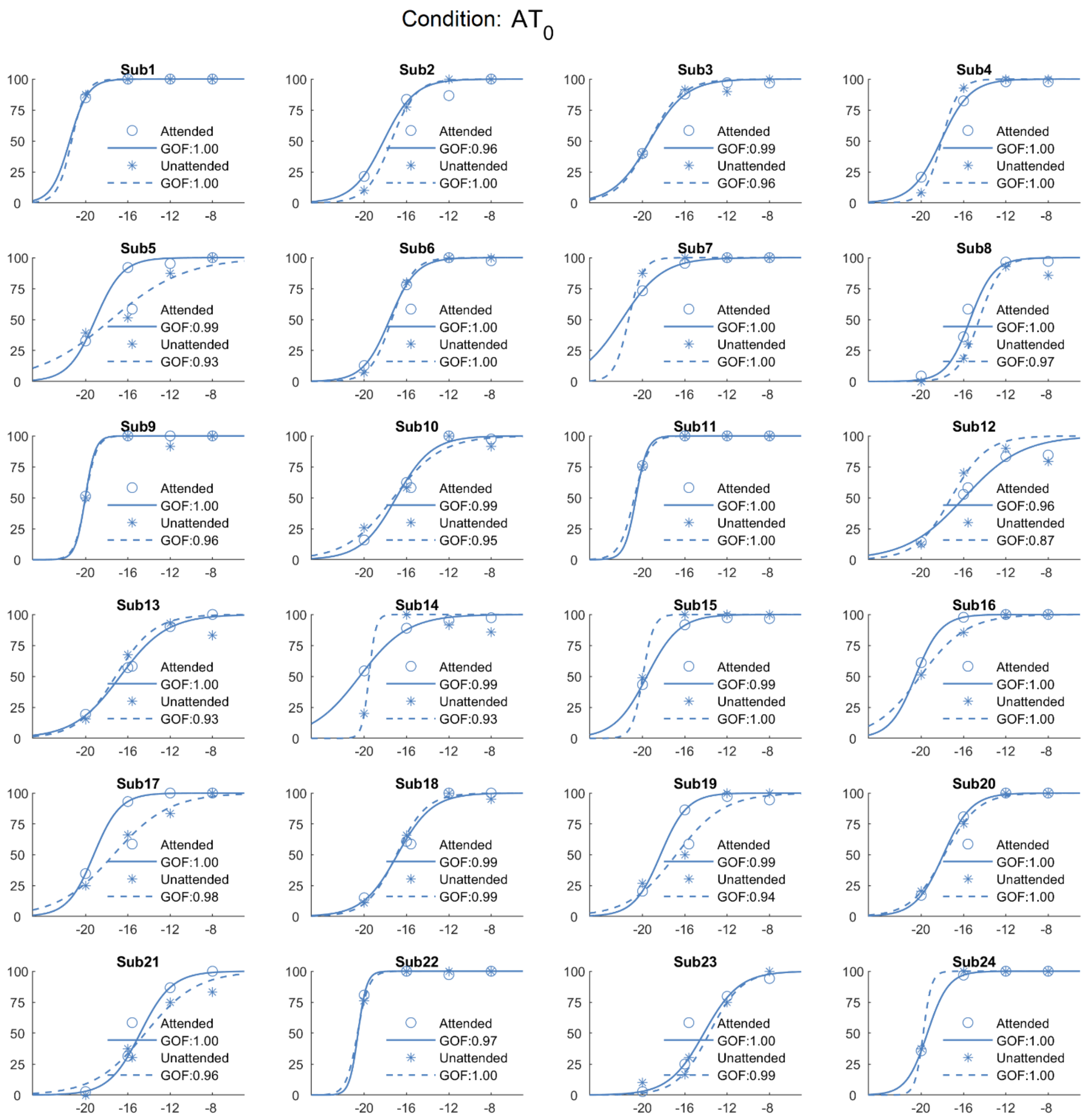

Supplementary Figure 6. *Same as Figure S5, but for the in-phase audio-tactile condition.*

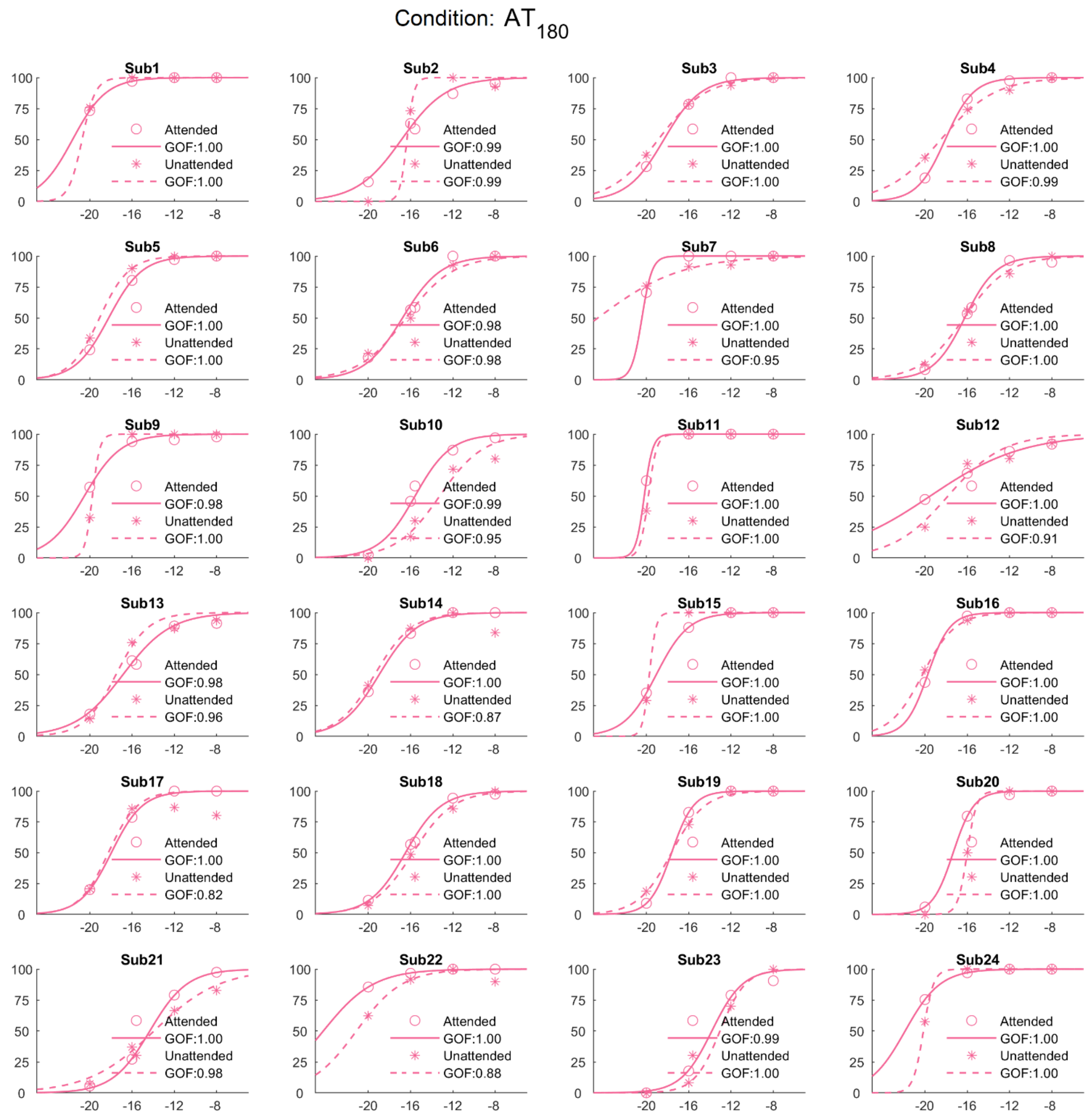

Supplementary Figure 7. *Same as Figure S5, but for the anti-phase audio-tactile condition.*

Reference

Allison, T., McCarthy, G., & Wood, C. C. (1992). The relationship between human long-latency somatosensory evoked potentials recorded from the cortical surface and from the scalp. *Electroencephalography and Clinical Neurophysiology/Evoked Potentials Section*, *84*(4), 301–314. https://doi.org/10.1016/0168-5597(92)90082-M

Bottini, G., Paulesu, E., Sterzi, R., Warburton, E., Wise, R. J. S., Vallar, G., Frackowiak, R. S. J., & Frith, C. D. (1995). Modulation of conscious experience by peripheral sensory stimuli. *Nature*, *376*(6543), 778–781. https://doi.org/10.1038/376778a0

Scrivener, C. L., & Reader, A. T. (2022). Variability of EEG electrode positions and their underlying brain regions: Visualizing gel artifacts from a simultaneous EEG-fMRI dataset. *Brain and Behavior*, *12*(2). https://doi.org/10.1002/brb3.2476
